# Dietary switch reveals fast coordinated gene expression changes in *Drosophila melanogaster*

**DOI:** 10.18632/aging.100662

**Published:** 2014-05-14

**Authors:** Rachel Whitaker, M. Pilar Gil, Feifei Ding, Marc Tatar, Stephen L. Helfand, Nicola Neretti

**Affiliations:** ^1^Department of Molecular Biology, Cell Biology, and Biochemistry, Brown University, Providence RI, 02912, USA; ^2^Department of Ecology and Evolutionary Biology, Brown University, Providence RI, 02912, USA; ^3^Center for Computational Molecular Biology, Brown University, Providence, RI 02912, USA

**Keywords:** Life span, dietary restriction, dietary switch, gene expression, Drosophila melanogaster

## Abstract

Dietary restriction (DR) reduces age-specific mortality and increases lifespan in many organisms. DR elicits a large number of physiological changes, however many are undoubtedly not related to longevity. Whole-genome gene expression studies have typically revealed hundreds to thousands of differentially expressed genes in response to DR, and a key open question is which subset of genes mediates longevity. Here we performed transcriptional profiling of fruit flies in a closely spaced time series immediately following a switch to the DR regime and identified four patterns of transcriptional dynamics. Most informatively we find 144 genes rapidly switched to the same level observed in the DR cohort and are hence strong candidates as proximal mediators of reduced mortality upon DR. This class was enriched for genes involved in carbohydrate and fatty acid metabolism. Folate biosynthesis was the only pathway enriched for gene up-regulated upon DR. Four among the down-regulated genes are involved in key regulatory steps within the pentose phosphate pathway, which has been previously associated with lifespan extension in *Drosophila*. Combined analysis of dietary switch with whole-genome time-course profiling can identify transcriptional responses that are closely associated with and perhaps causal to longevity assurance conferred by dietary restriction.

## INTRODUCTION

Dietary restriction (DR) extends longevity and delays the occurrence and progression of age associated diseases in a range of organisms [1-4]. The ubiquity of these effects suggests there should be conserved common molecular pathways underlying how animals slow aging in response to DR. Such mechanisms that might be elucidated in model organisms may therefore apply to mammals and even perhaps primates including humans [5, 6].

One approach to discover such underlying mechanisms of longevity assurance is to study age-dependent gene expression in DR relative to normal-diet animals.

Perhaps unsurprisingly, a great many genes are seen to differ between these groups and it is likely that only a fraction of these actually participate in the mechanisms that directly confer longevity assurance. The breadth of overall transcript changes in response to diet is illustrated by meta-analysis of publicly available transcriptional studies. Swindell compiled 40 DR gene expression cases in mouse and identified 12,214 differentially expressed genes [7]. Plank et al. [8] performed a meta-analysis of caloric restriction experiments in mammals as a class and identified common pathway signatures affected by DR, including growth hormone signaling, lipid metabolism, immune response, retinol metabolism, copper ion detoxification, and circadian. Wuttke et al. [9] utilized an even larger set of animals and identified evolutionarily conserved DR gene network, finding that DR commonly suppresses translation and stimulates an ancient reproduction-related process. Fewer analyses of chronic differences between DR and normal food have been conducted with Drosophila. Pletcher et al. [10] examined the chronic effect of DR with samples taken at six and eight different age points in the control and DR cohorts respectively over the course of their lifespan. This design identified 2,079 genes whose transcript abundance associated with adult diet, with down-regulated genes primarily involved in cell growth, metabolism, and reproduction.

An alternative approach builds from analysis of the acute response of transcript levels to a shift in nutrient uptake. Initially this was applied to wholesale shifts in nutrient quality such as acute fasting or removing all dietary protein. Zinke et al. [11] studied second instar Drosophila larvae when starved or when fed only sugar with samples taken at 1, 4, and 12 hours. Based on a criterion where a 4-fold change occurred in at least one time point, 40 genes were regulated upon starvation, but not in sugar conditions, and 51 genes were regulated in sugar condition, but not under starvation. Gershman et al. [12] studied the global gene expression dynamics at high temporal resolution in response to nutrition in Drosophila but only looked at complete yeast deprivation with acute replacement. Approximately 3,500 transcripts responded to nutrition, most notably in the insulin and target of rapamycin pathways, purine synthesis, TCA-biosynthetic functions and mito-chondria biogenesis.

Besides rapidly adjusting transcript profiles to acute changes in diet, diet switches rapidly alter age-specific mortality. When switched from protein to non-protein diets, the age-specific mortality of formally protein-fed adults quickly adopts the mortality rate and trajectory of a continuously non-protein-fed cohort [3, 13, 14]. Remarkably, when flies are shifted from a rich diet to just a relatively restricted diet, within days the cohort adopts the same trajectory of low age-specific of adults continuously maintained on restricted diet (and vice versa for cohorts switched from restricted to rich diets) [3, 13, 14]. These observations suggest that the molecular, cellular and physiological changes caused by DR to extend lifespan must occur within a short time frame after adults experience an alternative diet. Fine scale time series analysis of transcript responses in this window may thus reveal genes that are responsible for the mechanisms of DR longevity assurance. In this report we integrate the fine-scale time series approach of transcriptome analysis with diet switch demography. We quantify mRNA by sequencing from flies immediately following a rich-diet to restricted-diet switch until the time at which the switched cohort acquires the mortality trajectory of its continuously restricted diet control. As reference points we likewise quantify mRNA at each time point from the continuously rich- and restricted-cohorts.

## RESULTS

### Mortality rapidly acquires a restricted diet trajectory upon switch in diet

To identify the potential mechanism most proximally responsible for reduced mortality conferred by DR we applied an experimental protocol where adults are acutely switched from a full diet (control food; CF) to a restricted diet (restricted food; RF). Control food consists of sucrose, yeast and agar, while restricted food contains the same ingredients but with only 1/3 of the sucrose and yeast.

**Figure 1:**
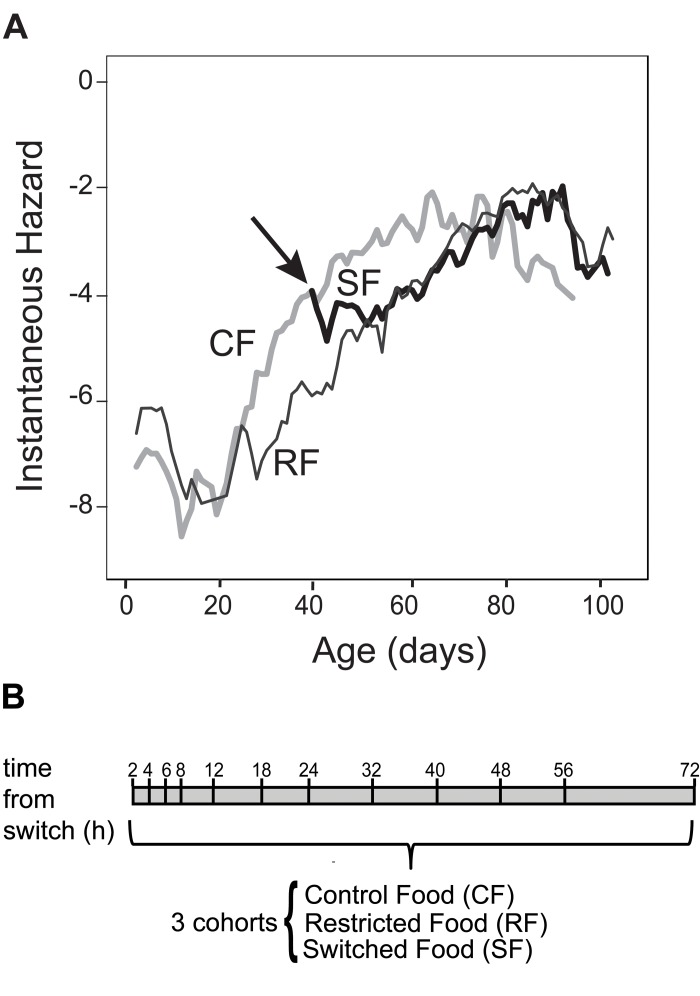
Rationale, experimental design and switch in mortality rate (**A**) Age-specific mortality rates of female Drosophila vary according to dietary levels. Female flies cultured in mixed-sex populations given control food (CF) cohort show an acceleration of age-related increases in mortality rate as compared to females on restricted food (RF) cohort. The flies switched from control food to restricted food (SF) show a deceleration in age-specific mortality rate behaving as the RF cohort. (**B**) Schematic of the experimental design. Canton-S flies were aged for 40 days in a CF or RF diet as described in Materials and Methods. On day 40, half of the flies on the CF diet were switched to the RF diet. The time line (hours) is shown on the top. Total RNA was extracted from head/thorax at the indicated time points from CF, RF and SF cohorts. The processed RNA was hybridized to Affymetrix Drosophila 2.0 arrays

In previous reports, switching adult Drosophila from a full diet to a diet that is diluted for both yeast and sugar causes the mortality rate of the switched cohort to adopt the mortality level and trajectory of a continuously restricted cohort within 2 days [3, 13]. Here, we determined the mortality dynamics of a cohort that is switched between CF and RF. Adult Canton-S flies were collected within 24 hours upon eclosion window and distributed into vials with 25 males and 25 females. 240 vials were assigned to control food (CF cohort). 120 vials were placed on restricted food (RF cohort). Each day, flies were passed onto new food, and dead flies were removed and counted. Mortality rates were estimated daily to determine the time at which there was a measurable separation between the mortality trajectories of the CF and RF cohorts [15]. This appeared at day 40 the number of deaths in both cohorts was sufficiently large to reveal a consistent 1.5-fold difference in age-specific instantaneous mortality rate between cohorts. Accordingly, at day 40, 120 vials were switched from control food to restricted food (Switch Food (SF) cohort). Age-specific mortality of the SF cohort dropped to the level and trajectory of the RF cohort within 3 days (Figure 1A and S1). Our diet switch dynamics based recapitulates the rapid mortality switch reported previously [3, 13].

### Transcriptional profiling of diet switch

Transcriptional changes that contribute to reduced mortality during DR must occur within the time-frame when a switched cohort adjusts its mortality to the level of the continuous RF cohort, that is within the three days following the acute diet switch. Hence we profiled gene expression in the CF, RF and SF cohorts at high resolution within the first 72 hours following the diet switch. Beginning at age 40 days we sampled 25 females and 25 males from each of at least 3 vials from each cohort (recording these decrements as censored observations in the life table) at 2, 4, 6, 8, 12, 18, 24, 32, 40, 48, 56, 72 hours (Figure 1B). mRNA was extracted from head and thorax of female flies for transcriptional profiling based on Affymetrix Drosophila 2.0 microarrays.

### Transcript differences among continuously control-fed and restricted-fed cohorts

853 genes were differentially expressed between the CF and RF cohorts as measured across all time points between ages 40 to 43 days. These genes represent all the cumulative and secondary differences produced by 40+ days upon different diets as well as any differences that were required to produce or maintain mortality differences among these cohorts. The magnitude of these changes ranged between 0.04-fold for Lsp2 to 28.79-fold for CG8147, with most (62.6%) cases representing reduced expression. Fifteen KEGG pathways (see Methods) are significantly enriched among these differentially expressed genes [16] (Table 1). Down-regulated genes were particularly associated with pathways of carbohydrate and fatty acid metabolism. Pathways associated with up-regulated genes included folate biosynthesis, ubiquinone/terpenoid- quinone biosynthesis, and oxidative phosphorylation. Gene Ontology (GO) analysis of biological processes provides a broader platform for classification that recapitulates the KEGG outcomes and identifies up-regulated genes in the categories of proteolysis, cellular respiration and electron transport chain, and down-regulated genes in categories such as lipid modification, multicellular organismal aging and triglyceride homeostasis (Table S1).

**Table 1:** KEGG pathways enriched for down- and up-regulated genes in RF versus CF

Pathway	q value¶	#genes
**Genes Down-regulated**		
Galactose metabolism	2.04E-06	11
Valine, leucine and isoleucine degradation	2.68E-04	9
Fatty acid metabolism	0.001359	8
Fatty acid elongation in mitochondria	0.003086	4
Starch and sucrose metabolism	0.003432	10
Metabolism of xenobiotics by cytochrome P450	0.003432	10
Pentose and glucuronate interconversions	0.004195	8
Pentose phosphate pathway	0.004715	6
Pyruvate metabolism	0.010222	8
Drug metabolism - cytochrome P450	0.010382	9
Glutathione metabolism	0.012249	9
Fructose and mannose metabolism	0.020554	6

**Genes Up-regulated**		
Oxidative phosphorylation	1.70E-10	28
Folate biosynthesis	0.004233	7
Ubiquinone and other terpenoid-quinone biosynthesis	0.005484	4

¶ The Benjamini and Hochberg method was used for Multiple Hypothesis Test correction allowing a maximum value cutoff of 0.05.

### Patterns of transcript dynamics upon diet switch

**Figure 2:**
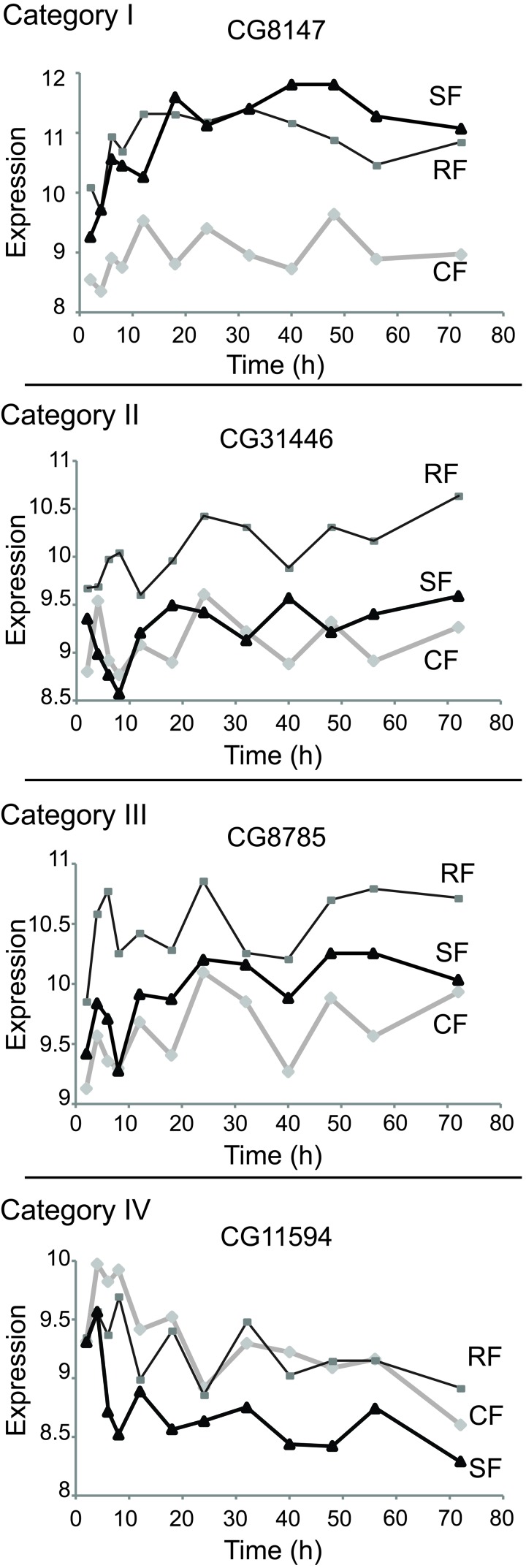
Expression plots of selected genes in each category Shown are examples from each consistent category. Represented in abscises are time points (hours) after switch, in ordinates are shown the mRNA expression scores in the log2 scale

From the 853 genes that differentiate the CF and RF cohorts, genes likely to cause reduced mortality in DR flies will show temporal dynamics in the SF cohort. These candidate DR affecter genes are expected to depart from the expression level of the CF cohort and acquire the expression level observed in the RF cohort. In a statistical test these Category I switching genes show no significant difference between SF and RF but a significant difference between SF and CF (based on FDR threshold of 0.05, see Methods). 144 genes produced Category I switching dynamics, illustrated by the case of CG8147 in Figure 2. Category II and III contain genes whose expression level is determined by the nutritional history of the cohort. Category II genes (198 cases, Table 2) retained the expression level of the CF cohort after the time of the diet switch (at all time points: SF = CF and SF ≠ RF), exemplified by CG31446 in Figure 2. Category III genes (7 cases, Table 2) diverged from the CF cohort expression level after the switch but did not acquire the expression level of the RF cohort within the mortality switch timeframe; exemplified by CG8785 (Figure 2). Category IV genes were identified from those that did not differ between RF and CF (not in the set of 853); among these, 12 genes in the SF cohort diverged from the level measured in CF but did not adopt the level seen in RF, exemplified by the CG11594 gene (Figure 2). These *deviating genes* respond to the diet switch but not to chronic DR.

**Table 2:** Distribution of genes in the four main classes of temporal responses to the switch in diet

Categories	Behavior	Interpretation	# Genes	# GO¶	# KEGG¶
**I**	diet dependent & diet responsive & switching	SWITCHING GENE: Response consistent with mortality change	144 99↓ 45↑	110 82↓ 28↑	55 45↓ 10↑
**II**	diet dependent & diet unresponsive & non-switching	REFRACTORY GENE: Level depends on past nutritional history	198 53↓ 145↑	123 34↓ 89↑	48 16↓ 32↑
**III**	diet dependent & diet responsive & non-switching	RESPONSIVE GENE: Response not consistent with mortality change	7 3↓ 4↑	5 3↓ 2↑	3 2↓ 1↑
**IV**	diet independent & diet responsive & non-switching	DEVIATING GENE: Responds to change but not to diet level	12 8↓ 4↑	10 7↓ 3↑	3 2↓ 1↑

¶ The numbers of genes found in GO categories and KEGG pathways correspond to unique non-redundant genes as obtained from FlyMine.

Of the original 853 differences between CF and RF, only 349 genes met the statistical criteria to be assigned to Categories I, II or III. The remaining genes displayed statically indeterminate behavior. Some of these cases, for instance, switched over the course of the entire 72 hours (Figures S2) but too slowly, with too little amplitude or with too much variability to meet statistical significance. These genes are assigned to Categories VI, VII and VIII according to the criteria described in Table S2. All remaining genes that were not significantly different between any of the diet cohorts were assigned to Category V (Table S2).

### Pathway enrichment analysis

Categories I, II, III and IV are associated with distinctively enriched pathways, based on the same methodology applied to chronic DR genes. Fifty-five genes of Category I were present in annotated KEGG pathways while 110 were associated with at least one gene ontology. In both classification systems enrichment was most prominent for pathways of carbohydrate and fatty acid metabolism. In particular, down-regulated pathways include several observed for chronic DR, including pentose phosphate pathway, glycolysis/gluconeogenesis, and fatty acid metabolism (Table 3). In contrast, genes of Category I were up-regulated in only a single KEGG pathway, folate biosynthesis. GO analysis recapitulated most of the KEGG findings and further identified oxidation reduction process, oxoacid metabolic process, NADPH regeneration, and NADP metabolic process as enriched for down-regulated genes. Switching genes in the folate biosynthesis pathway include CG10592, CG3290, CG5150, CG8147, which function together to convert 2-amino-4-hydroxy-6-(erythro-1,2,3-trihydroxypropyl)-dihydropteridine triphosphate to dihydroneopterin. Interestingly, all proteins produced by these genes are alkaline phosphatases and show some of the largest levels of up-regulation in both chronic DR and in response to the diet switch (Figure S4B-E). No other alkaline phosphatases were detected as having a similar behavior (Figure S4A).

**Table 3:** - KEGG pathways enriched for genes in each of the four main classes

Pathway	q value¶	#Genes

**Category I: Genes Down-regulated**		
Pyruvate metabolism	0.001044	7
Pentose phosphate pathway	0.001687	5
Fructose and mannose metabolism	0.007189	5
Glycolysis / Gluconeogenesis	0.041722	5
Fatty acid metabolism	0.043732	4
Galactose metabolism	0.047511	4
**Category I: Genes Up-regulated**		
Folate biosynthesis	2.03E-04	4

**Category II: Genes Down-regulated**		
Galactose metabolism	0.001854	4
Starch and sucrose metabolism	0.002988	5
Fatty acid elongation in mitochondria	0.03675	2
**Category II: Genes Up-regulated**		
Oxidative phosphorylation	3.48E-05	11
		
**Category III: Genes Down-regulated**		
No enrichment found		
**Category III: Genes Up-regulated**		
No enrichment found		
		
**Category IV: Genes Down-regulated**		
Organic cation transport	0.018776	2
Organic cation/anion/zwitterion transport	0.018776	2
**Category IV: Genes Up-regulated**		
No enrichment found		
		

¶ The Benjamini and Hochberg method was used for Multiple Hypothesis Test correction allowing a maximum value cutoff of 0.05

Forty-eight genes of Category II belonged to at least one KEGG pathways, and 123 fell within to at least one gene ontology. Down-regulated genes again occurred in pathways of carbohydrate and lipid metabolism, such as Mal-A1, Amyrel and CG6543. These are notably distinct from the pathways enriched for switching genes. And again, only one pathway was observed to contain up-regulated genes, in this case, oxidative phosphorylation, which was also found to be enriched for up-regulated genes in chronic DR. KEGG lists 28 genes within the Drosophila oxidative phosphorylation pathway; 11 of these were up-regulated Category II genes while the remaining 17 fell in uninformative Categories VI and did not demonstrate any switching behavior (Supplemental Data File 2). The oxidative phosphorylation pathway is enriched for genes that are up-regulated in chronic DR, but does not contain any switching genes. GO analysis recapitulated all the inferences from the KEGG pathways but further identified proteolysis as a category enriched for up-regulated refractory genes (Table S3).

Categories III and IV in general contain too few genes to provide statistical power for pathway enrichment analysis. It is therefore notable that enrichment with down-regulated genes with the organic cation transport pathway (KEGG) and trans-membrane transport (GO) was detected for Category IV.

**Figure 3:**
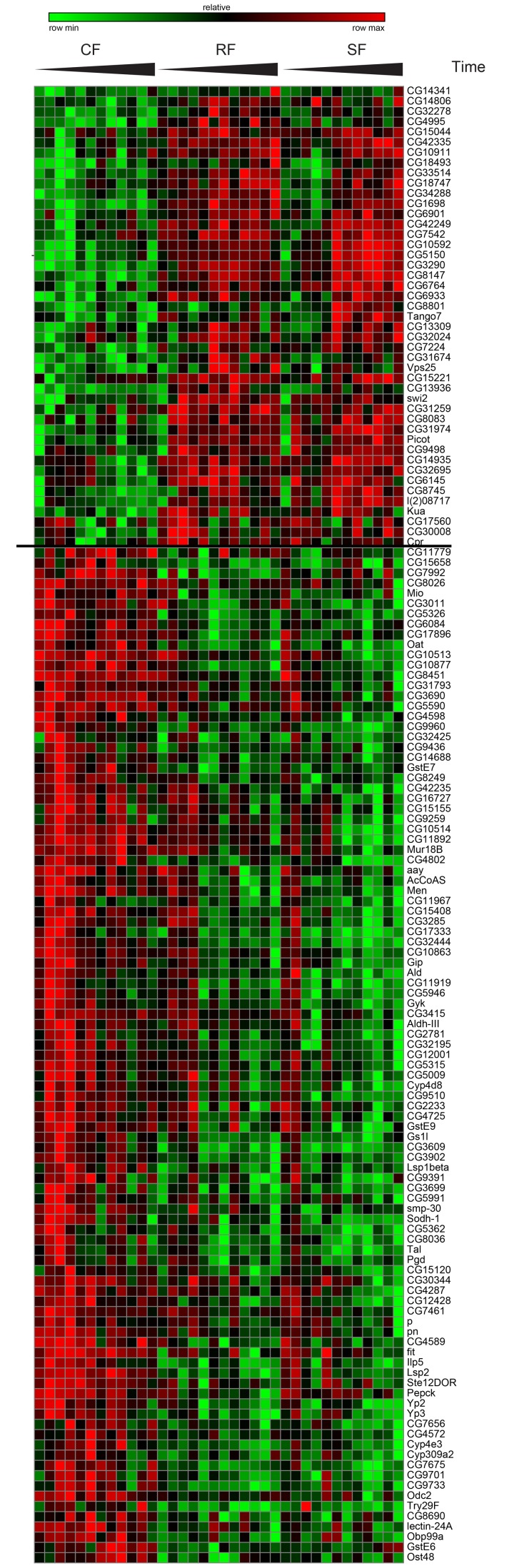
Gene expression of switching genes Hierarchical clustering for Category I genes. The two resulting clusters are shown in a heat map of log2 expression values with green corresponding to low expressed genes and red to high expressed genes

**Figure 4:**
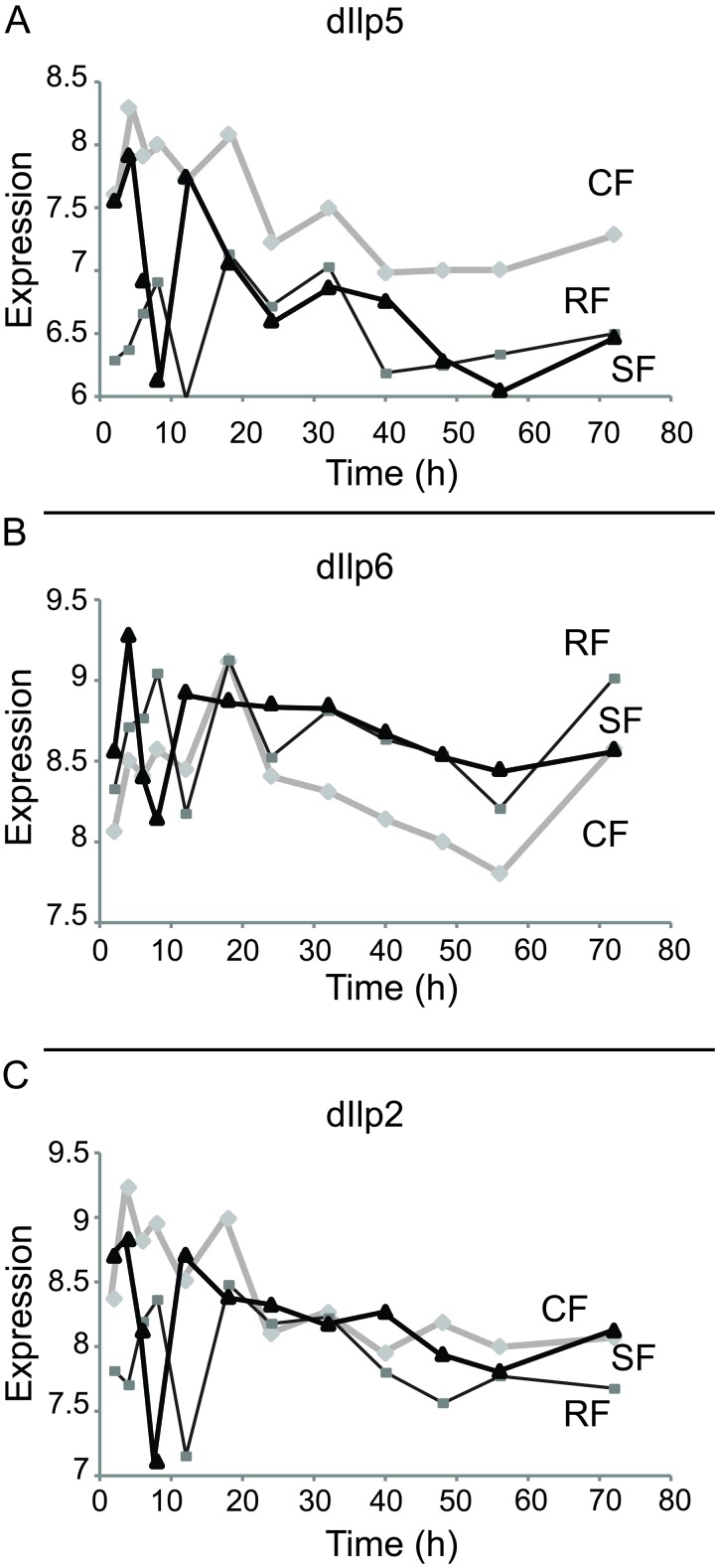
Expression of Drosophila insulin-like peptide in each cohort Represented in abscises are time points (hours) after switch, in ordinates are shown the mRNA expression scores in the log2 scale. (**A**) Dilp5 is a category I gene, with expression down-regulated in RF cohort as compared to CF cohort. (**B**) Dilp6 is up-regulated in RF cohort as compared to CF cohort. However, dilp6 can't be clearly assigned to one of the main category due to its intermediate behaviors during time courses. (**C**) Dilp2 level doesn't change between three cohorts

Reduced insulin/IGF signaling extends life span in many species including Drosophila [17]. The Drosophila genome encodes 8 insulin-like peptides (*dilp1-8*). Ablation of DILP producing cells within adults extends lifespan, as does mutation of *dilp2*. Although single mutations of *dilp2* or *dilp5* do not affect the ability of DR to extend lifespan, the combined mutation of dilp2, 3, and 5 extends lifespan and appears to block the impact of DR upon aging [18]. Probes for *dilp2*, *dilp5* and *dilp6* were present on the Drosophila 2.0 microarrays. The level of mRNA for *dilp2* was constant and similar amongst all the cohorts (Category VI, Figure 4C). *dilp5* presented as a switching gene (Category I, Figure 4A). Although *dilp6* was elevated in chronic DR, the variance of its expression level was too large to gain statistical significance as a Category I gene even though expression level in the SF cohort appears to match that of the RF cohort at late time points (Figure 4B).

The TOR pathway mediates at least part of the longevity response of DR [19-22]. 23 of the 26 genes in the TOR pathway (KEGGID 04150) were present on the microarray, and some of these were represented by multiple probesets for a total of 27 probesets. All probesets in the TOR pathway belonged to Category V (Supplemental Data File 2), i.e. they were not significantly different between any of the diet cohorts. We conclude that in our experiment effects of DR on the TOR pathway genes must occur post-transcrip-tionally and could not be detected by microarrays.

### Regulatory elements in the promoters of switching genes

Many Category I genes presented remarkably quick dynamics, switching either up (45) or down (99 cases) within 4-6 hours of the change in diet. Several mechanisms could contribute to these responses, including induction or repression at promoters by transcription factor complexes, transcription initiation of stalled promoter complexes, and mRNA degradation by microRNA.

To identify potential regulatory elements and transcription factors that might coordinate this rapid switching response, we performed an *in silico* promoter analysis for conserved motifs. Over-expressed and under-expressed switching genes were curated into groups, conditioned on criteria of complete switch with 6-8 hours. This criterion produced a set of 18 genes with increased expression and 10 with reduced expression (Table S4). The FASTA repeat-masked sequences for the promoters of these genes was restricted to a region from −2000 to −1 from the most 3’ TSS to reduce overlap with neighboring genes. Gibbs analysis [23] revealed significant enrichment for WWTTTAATTR and VCWGCTGATY among the up-regulated genes (Figure S3A and S3B), and GTNATTNGTTTG and ANANACATNTTTNW among the down-regulated genes (Figure S3C and S3D).

Based on the Transfac and Jasper databases for regulatory factors [24, 25], the WWTTTAATTR motif corresponds to the binding site of the protein Lmx1b (q = 0.01), a LIM homeodomain transcription factor involved in the development of the brain and spinal cord. The other three motifs did not match any transcription factor in the two databases.

Promoter sequences were further analyzed using MatInspector [26], which uses a large library of position weight matrices for transcription factor binding sites to locate matches in DNA sequences. Five factor matrix matches were found in promoters of switching genes with increased expression with DR – Spalt-like transcription factor, PREB core-binding element, Autoimmune regulatory element binding factor, MEF3 consensus sequence binding factor, PR domain zinc finger protein 14 – and two for the down-regulated genes – Cyclin D binding myb-like transcription factor, and Zinc finger transcription factor RU49.

### Contrasting effects between fasting and diet switching upon gene expression

**Figure 5:**
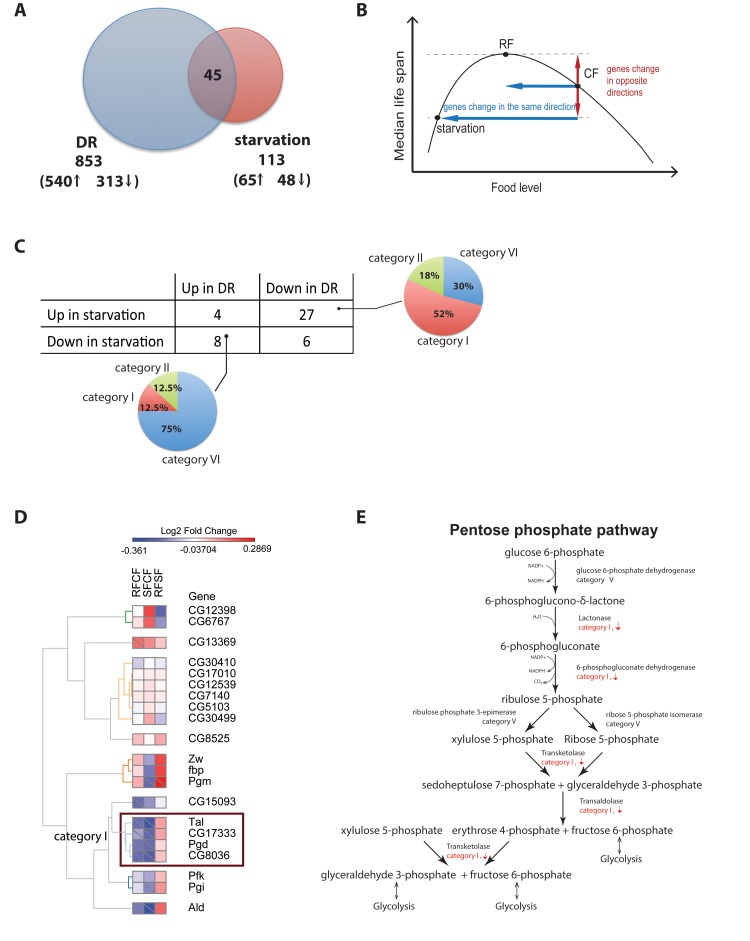
Different gene regulation between Dietary restriction and starvation (**A**) Comparison of dietary restriction and starvation in D. melanogaster. The intersection between both dietary interventions show a small number of genes (44) with most of them changing in opposite directions in starvation and dietary restriction. (**B**) Proposed model. As food level changes, genes that are change in the same direction for both starvation and Dietary Restriction are likely not involved in lifespan. However, genes that change in opposite directions in starvation vs. dietary restriction correlate with lifespan. (**C**) Contingency table of genes up- and down-regulated in chronic DR and starvation and their distributions in the different categories of responses to the diet switch. (**D**) Heatmap of the fold-changes between the three cohorts for all gene annotated in the KEGG pentose phosphate pathway. Cells marked with a diagonal bar correspond to genes and comparisons that reached statistical significance. (**E**) Transcriptional response to the diet switch of genes within the core steps in the pentose phosphate pathway

An acute diet shift is likely to produce some rapid transcriptional changes required for metabolic adaptations independent of those responsible for reduced age-specific mortality. Although we cannot distinguish these classes within our current data, some insight is gained by comparing genes from our categories to transcriptional responses reported in cases for Drosophila adults acutely subjected to fasting, which typically decreases their lifespan. We would expect genes that mediate the longevity response to change in opposite directions in response to DR and starvation (Figure 5B).

Fujikawa et al. examined the gene expression changes in flies starved for 24 hours and observed 65 genes whose expression increased under starvation, and 48 genes whose expression decreased [27]. In both our chronic DR and Fujikawa *et al.* experiments the number of up-regulated genes exceeded the number of down-regulated genes although in slightly greater proportion in chronic DR (172% in DR versus 135% in starvation). Of the 853 genes differentially expressed in our study in response to chronic DR, 45 were also detected as differentially expressed in response to starvation (Figure 5A). Only 22% (i.e. 10 genes) changed in the same direction in both experiments, and of these, 4 genes were up-regulated in both experiments (CG1662, CG18135, and klu in category VI and CG16986 in category 2), and 6 were down-regulated in both experiments (CG14688, CG32425, CG943, fit, and Lsp2 in category I and sug in category VII) (Figure 5C). Most genes differentially expressed in both experiments, 78% (i.e. 35 genes), changed in opposite directions. Of these, a minority (8 genes) were up-regulated in DR but down-regulated in starvation and belonged primarily to category VI. The remaining 27 genes were up-regulated in DR and down-regulated in response to starvation, and more than half of them (14 or 52%) were categorized as switching genes. Pathway analysis performed on this group of 14 switching genes showed that the pentose phosphate pathway and pyruvate metabolism were significantly enriched (q value < 0.05). In particular a closer inspection of the pentose phosphate pathway revealed that a group of four key enzymes regulating several steps along most of the pathway – lactonase, 6-phosphogluconate dehydrogenase, transketolase, and transaldolase – were significantly down-regulated in response to both adult onset and chronic dietary restriction, but significantly up-regulated in response to starvation (Figure 5D-E). Fasting will increase the flux of glucose 6-phosphate to ribose 5-phosphate, and the production of NADPH+H and reduce glutathione, while DR will retard this flow.

## DISCUSSION

Gene expression profiling of dietary restriction has consistently identified hundreds to thousands of differentially expressed genes in most model organisms [7-10, 28]. This large number demonstrates the wide set of physiological changes elicited by DR, and at the same time, poses a challenge in identifying the specific subset of genes responsible for mediating the longevity response. We developed a novel experimental design to narrow down this large list of genes to a smaller set of candidates with a more direct association to longevity. Our design relied on the striking demographic response of adult *Drosophila melanogaster* to DR [3, 13, 14]. We confirmed that when adult fruit flies on control food are switched to restricted food their mortality rate drops rapidly to the same level of flies kept on chronic DR. Because this drop occurs in a short window of 24-72 hours, we performed expression profiling at high resolution within this time window.

As expected, many genes (853) were differentially expressed in response to chronic DR. The overlap between our gene list and previously published data [29] was highly significant (35% overlap, hyper-geometric test p < 10^−27^), and dissimilarities were likely due to the different ages of flies in the two studies (40 days in the current study as opposed to 10 days in [29]) as previously reported [30]. A large number of pathways, mostly associated with carbohydrate and fatty acid metabolism, were enriched for down-regulated genes. Notably the GO category “aging” was also enriched for such genes. Because the “aging” gene ontology is comprised of genes associated to aging in previous studies, this finding is not surprising and attests to the fact that DR impacts a large fraction of these genes. Only a handful of pathways were enriched for up-regulated genes. Most notably among the latter group was oxidative phosphorylation, consistently with what has been previously reported [31], as well as proteolysis, indicating a possible increase in protein turnover in flies on chronic DR.

To investigate which genes respond to the diet switch we compared gene expression in the diet switched cohort to the control food and chronic DR cohorts, and identified four major classes of behaviors. Interestingly, only 17% of the 853 chronic DR genes showed a clear switching behavior, i.e. their pattern of expression showed a complete switch to the level observed in the chronic DR cohort within 72 hours of the change in diet. Most of these transitions were rapid and completed in as little as 4-6 hours. Since the demographic response to adult onset DR is completed within 72 hours of the diet switch, we propose the switching genes we identified are enriched for genes that directly modulate the longevity response to DR. Of the remaining genes, 23% of the chronic DR genes were not affected by the adult onset DR (refractory genes), and could be important in maintaining DR or cause other effects associated with DR, such as changes in fertility and behavior. The remaining chronic DR genes either changed their expression to a level that was different from both of the cohorts kept on constant food, displayed a slow and partial switch, or had too much variability in their expression pattern and could not be unambiguously assigned to either of the preceding categories.

Because of the rapid coordinated behavior of the switching genes we performed a motif search in their promoters to identify cis- and trans-acting elements that could potentially modulate their collective response. Motif finding based on a library of known transcription factors identified several candidates, mostly for up-regulated switching genes. De-novo motif finding identified primarily motifs that did not have any known associated transcription factors, with the notable exception of the LIM homeobox transcription factor 1-beta (Lmx1b), which is conserved across a broad range of species. Interestingly, in humans a member of the same family, Lmx1a, binds to the promoter of the insulin gene and stimulates its transcription [32]. These results highlight the potential for co-regulation of the switching genes through the action of transcription factors that respond to DR. Due to the rapidity with which switching genes respond, these transcription factors are likely modulated post-transcriptionally and their change in activity cannot be detected by transcriptional profiling.

The highest ranked down-regulated switching gene based on fold-change was Larval-serum protein 2 (Lsp-2), which belongs to the nutrient reservoir activities, synaptic target inhibition, and motor neuron axon guidance [33]. Interestingly, Lsp-2 was the only gene found to be significatively down-regulated in common between three related longevity-inducing interventions: DR, dSir2 overexpression and DN-Dmp53 expressing long-lived flies [28].

Within the insulin pathway, which has been extensively studies for its role in modulating lifespan, both DILP5 and DILP6 displayed a switching behavior, but in opposite directions, while DILP2 expression level did not show any dependence on the food level. Our results are consistent with those described in Bai et al., who have recently shown that overnight fasting increases the levels of the DILP6 mRNA in Drosophila fat body, while DILP5 mRNA expression decreases in the brain and DILP2 expression level remains unchanged [34]. Oxidative phosphorylation, one of the major pathways associated with longevity [31], although up-regulated under chronic DR, does not contain any switching genes. Within 72-hours after the switch, no genes in the oxidative phosphorylation pathway increased above the level on control food. This suggests that transcriptional changes in the oxidative phosphorylation pathway do not appear to be associated with the observed drop in mortality, although changes in the level of expression of genes in the oxidative phosphorylation pathway may switch after 72-hours and be important for maintenance of the longevity effects of chronic DR.

Another interesting finding is that genes in the folate biosynthesis pathway, particularly genes involved in the conversion of Dihydroneopterin to 7,8-Dihydro-pteroate, are up-regulated switching genes. While many of the enzymes mediating the folate biosynthesis pathway have not yet been identified in Drosophila [16], it is worth noting folate has been implicated in the aging process in several other organisms [35-37]. In particular, folate is a precursor to methionine, which is then converted into s-adenosyl methionine (SAM), a cell-wide donor of methyl groups. It is through this pathway that folate levels have been associated with DNA methylation levels and aging [38]. Additionally, folate is a source of carbon for the addition of methyl groups on DNA and histones, and folate deficiency can result in hypomethylation of DNA. Finally, mice that were fed a folate-deficient diet had disruptions in their circadian rhythms that resembled those of aged mice [39]. If the production of 7,8-Dihydro-pteroate is linked to the induction of folate biosynthesis in the more downstream part of the pathway as predicted, this induction could be a potential mechanism for up-regulation of methylation in the cell, and a potential mechanism for a fast response to dietary alterations, as suggested by recent work by Jiang *et al.* that demonstrated rapid increase in constitutive hetero-chromatin in response to DR [40].

Comparison of chronic DR genes with previously published data on fruit flies response to starvation gave us an opportunity to contrast two dietary reductions with opposite demographic effects (Figure 5B). The majority of those genes responding to both interventions showed changes in opposite directions, thereby demonstrating a more direct association with the demographic response than to the dietary change, which occurred in the same direction in both interventions. Most of the genes up-regulated in starvation and down-regulated in chronic DR were switching genes. In particular, four of these genes are involved in a set of key regulatory steps within the pentose phosphate pathway, suggesting that changes in flux in this pathway in response to DR might be associated with changes in mortality. Legan *et al.* reported that overexpression of glucose 6-phophate dehydrogenase (G6PD), the very first enzyme in the pentose phosphate pathway, extends lifespan in Drosophila melanogaster [41]. G6PD did not show any response to the diet switch and chronic DR in our study. This may reflect the positive effects of increasing glucose 6-phosphate dehydrogenase on oxidative stress, as suggested in [41] rather than an effect on the net metabolic flux through the pathway.

Although we demonstrate only a relatively small set of genes displays a complete switch in response to DR, our experimental design cannot discriminate which of these genes are necessary and sufficient for lifespan via DR. These genes could work cooperatively so that a coordinated change of many of them would be required to reduce mortality. It is also possible that chronic DR genes we classified as late switching or non-switching are important for maintaining the lower level of mortality beyond the 72 hours window. Nevertheless, this list of genes suggests a small set of genes and pathways that can be utilized to identify specific genes and pathways responsible for the mortality switch induced by DR and possibly important for longevity determination.

Our results indicate that the combination of a dietary switch and high-resolution expression profiling is a powerful paradigm to investigate the longevity response to DR and can identify a smaller subset of candidate genes that could modulate changes in mortality induced by DR.

## METHODS

### Fruit fly husbandry and conditions

Canton-S flies were obtained from the Bloomington Drosophila Stockcenter at Indiana University (Bloomington, IN) and kept at 25°C in a temperature-controlled incubator at 50% humidity with a 12-hour on/off light cycle. The parental generation of flies was raised on food containing 30 g/L autolysed yeast, 120 g/L sucrose, 50 g/L cornmeal, 10 g/L agar with added dry live yeast. Experimental flies were collected in a 24-hour window, anesthetized under light CO2, sorted into vials at a density of 25 males and 25 females per vial, and randomly divided into treatment groups. The flies were then passed every day on either Control Food (CF, 150 g/L sucrose, 150 g/L autolysed yeast, and 20 g/L agar, all w/v) or Restricted Food (RF, 50 g/L sucrose, 50 g/L autolysed yeast, and 20 g/L agar, all w/v) and the number of dead flies recorded. On day 40, half of the flies on Control Food were switched to the Restricted Food. During the course of the experiment, age-specific instantaneous mortality rate was analyzed and the separation of mortality rate between the food conditions was verified before the switch. Flies were sorted under light CO2 and collected at fixed time intervals 2, 4, 6, 8, 12, 18, 24, 32, 40, 48, 56, and 72 hours after the switch time point via snap freezing in liquid nitrogen and were stored at −80°C. Heads and thorax of female flies were collected for microarray experiments.

### Demographies

Demographies were performed on all experimental flies (total number of flies in demography: CF = 2892, RF = 2987, SF = 2278) and flies collected for microarray analysis were censored from the study on the day of their collection (total number of flies censored: CF = 1650, RF = 1825, SF = 1126). Log rank tests were performed using the survival package in R to obtain p-values. Maximum life span was calculated as the mean life span of the longest surviving 10% of the population. Instantaneous hazard and survivorship was computed via the “survival” R package (http://cran.r-project.org/web/packages/survival/).

### Gene expression studies

Total head and thorax RNA was isolated from at least 75 females using Trizol (Invitrogen) and further purified using RNeasy columns (QIAGEN). 5 μg total RNA was used with Affymetrix One Cycle DNA conversion Kit (Cat # 900431) and all steps were carried out according to the Affymetrix manual. Briefly, first RNA was converted to double stranded cDNA followed by a clean-up step using spin columns. The double stranded cDNA was amplified in an in-vitro transcription reaction overnight at 37 °C using Affymetrix IVT labeling kit (cat # 900449), resulting in biotin labeled cRNA. After clean-up of the labeled cRNA with spin columns, 15 μg of cRNA were fragmented using metal induced hydrolysis. 10 μg of the fragmented RNA were hybridized to Drosophila 2.0 arrays overnight at 45 °C, 60 rpm. The array was stained using Affymetrix Hybridization-Wash-Stain kit and Fluidics Script FS450_0002 on the Affymetrix 450 fluidics station and finally, the arrays were scanned using an Affymetrix 3000 G7 scanner. Probes were mapped to CG numbers using the drosophila2.db annotation package from Bioconductor. The data was PVAC filtered, quantile normalized and summarized using Robust Multichip Analysis (RMA) to obtain expression scores in the log2 scale. Data have been deposited in the Gene Expression Omnibus with accession number GSE47631. qPCR was used to validate a subset of genes and time-points and we observed a consistent correlation between qPCR and microarray trends (Figure S4).

### Data analysis

Paired t-test, two sided, was used to test for differential expression between RF and CF, SF and CF and SF and RF, using all time points. Points in each condition were paired for this test by their time of collection from the diet switch. A multiple testing correction was applied to the pooled p values from all tests and comparisons [42]. The False Discovery Rate (FDR) cutoff was set to 0.05 and genes were selected into different categories depending on q values between cohorts.

Heat map visualizations were conducted with GENE-E (http://www.broadinstitute.org/cancer/software/GENE-E/). Functional enrichment and pathway analysis were performed using Flymine [43], KEGG database [16], and the gene ontology (GO) term [44].

## SUPPLEMENTARY FIGURES, TABLES AND DATA FILES








