# Supplementary material for: Dietary switch reveals fast coordinated gene expression changes in Drosophila melanogaster
Source: Aging (Albany NY). 2014 May 14;6(5):355–68. doi: 10.18632/aging.100662 (PMC4069263; doi:10.18632/aging.100662)
Supplement: Supplementary file 1 [file aging-06-355-s001.pdf]

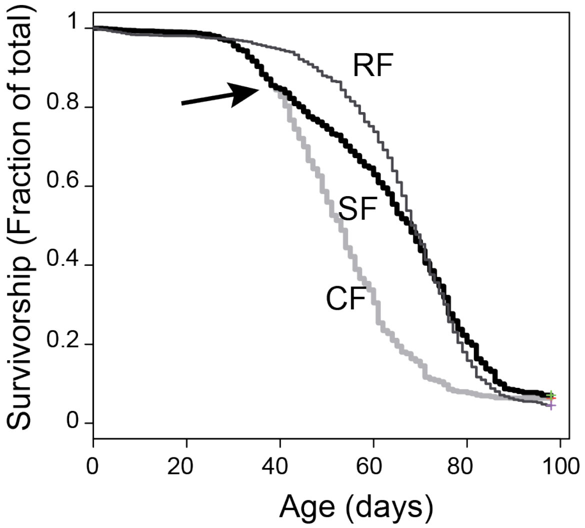
Survivorship (Fraction of total)
1
0.8
0.6
0.4
0.2
0
0
20
40
60
80
100
Age (days)
RF
SF
CF

Figure S1

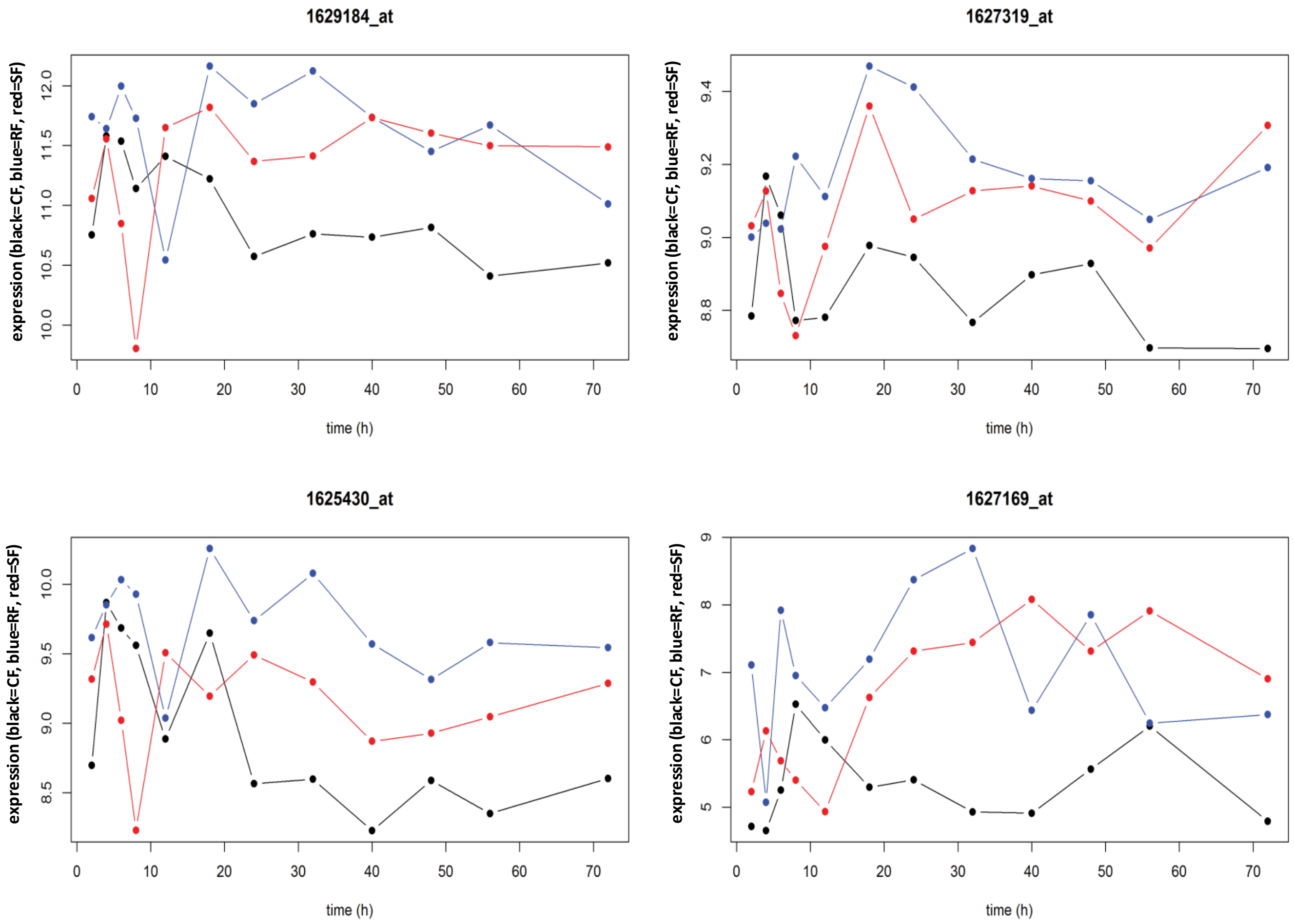
1629184_at
1627319_at
1625430_at
1627169_at
expression (black=CF, blue=RF, red=SF)
time (h)

Figure S2

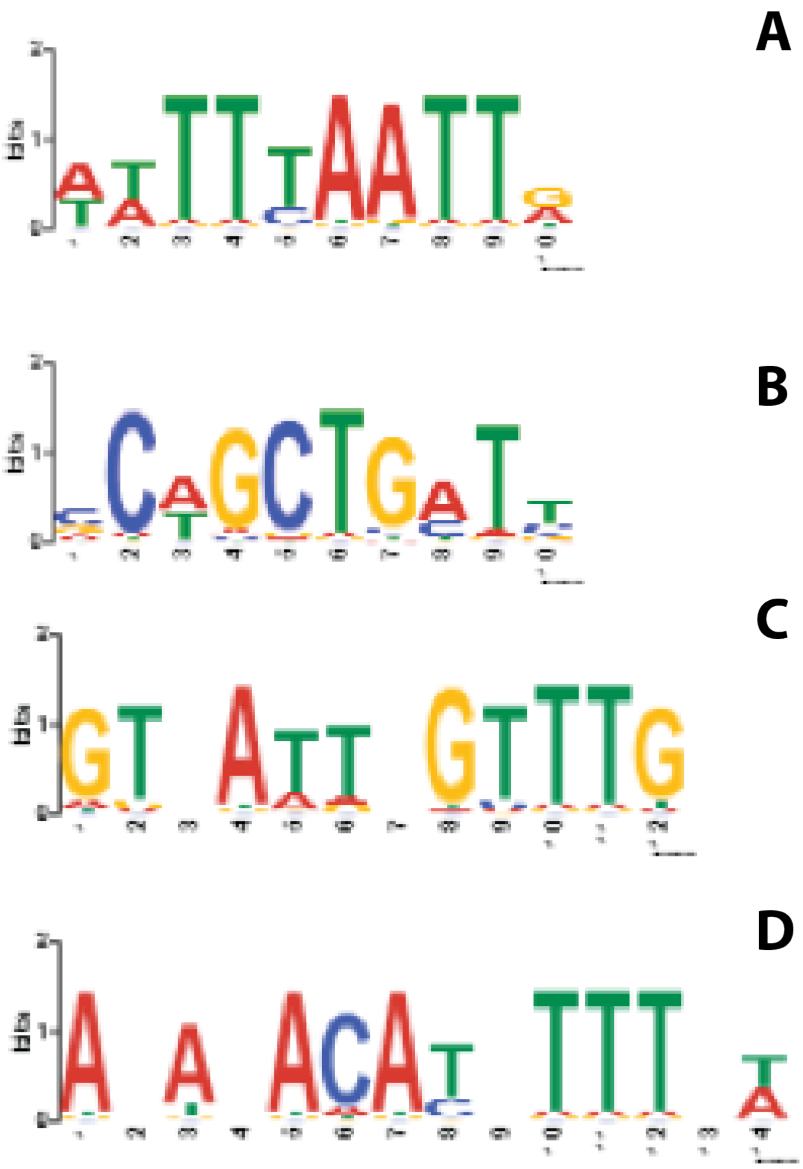

Figure S3

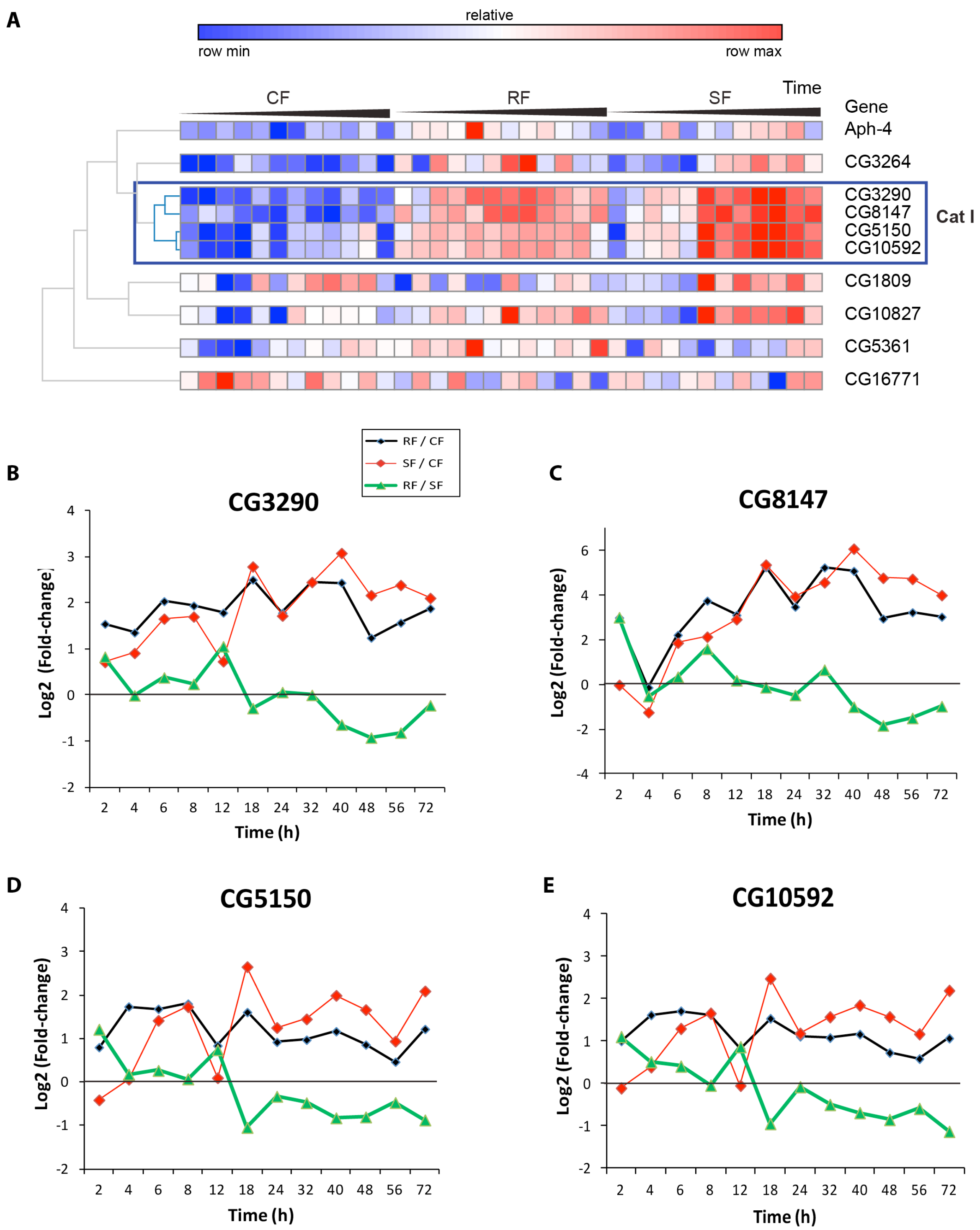

A
relative
row min
row max
CF
RF
SF
Time
Gene
Aph-4
CG3264
CG3290
CG8147
CG5150
CG10592
Cat I
CG1809
CG10827
CG5361
CG16771
RF / CF
SF / CF
RF / SF
B
CG3290
Log2 (Fold-change)
Time (h)
C
CG8147
Log2 (Fold-change)
Time (h)
D
CG5150
Log2 (Fold-change)
Time (h)
E
CG10592
Log2 (Fold-change)
Time (h)

Figure S4

MICROARRAY	qPCR (normalized to CF 2h)

CG8147

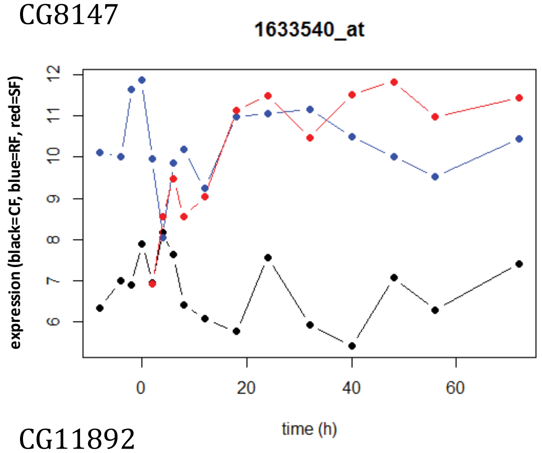

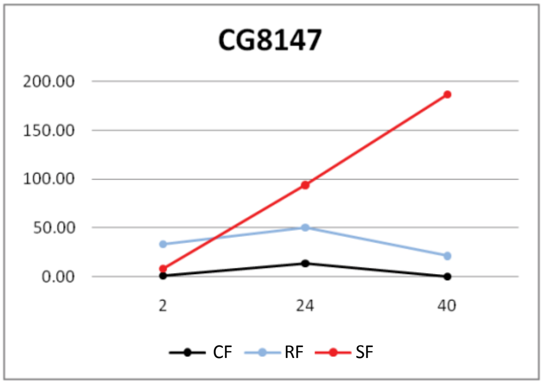

CG11892

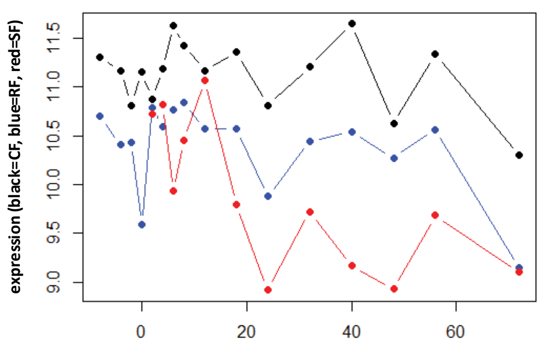

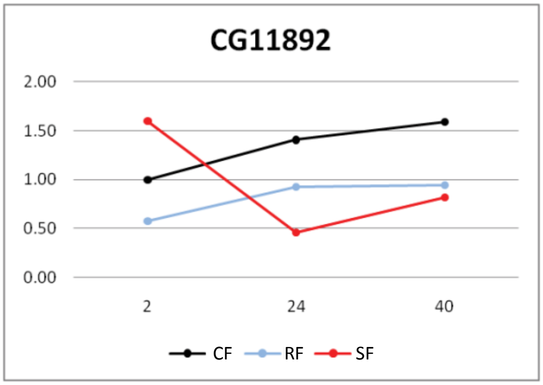

CG1698

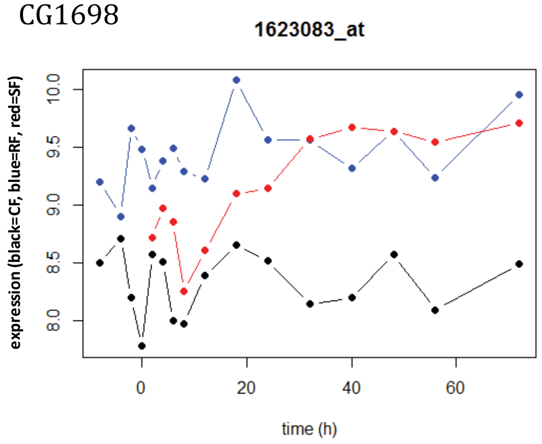

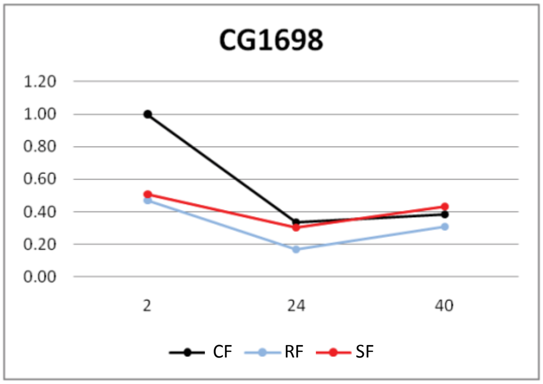

CG13947

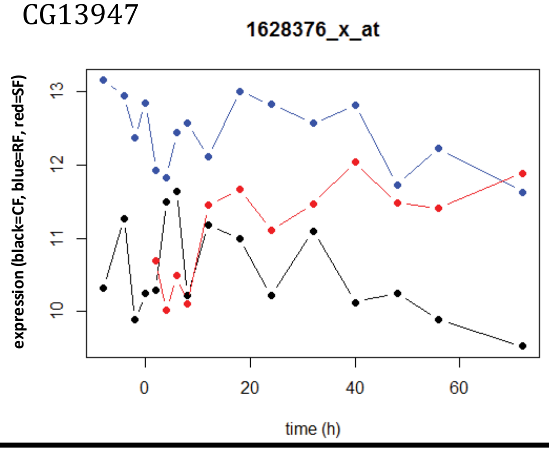

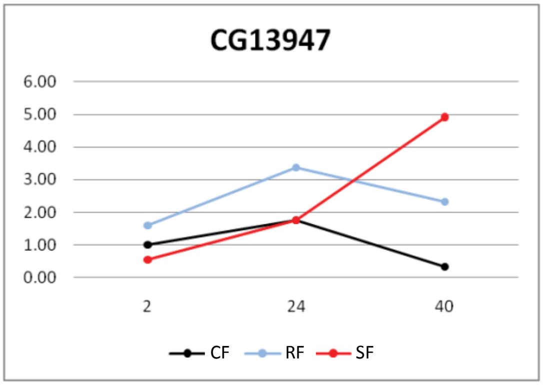

MICROARRAY	qPCR (normalized to CF 2h)

CG17100

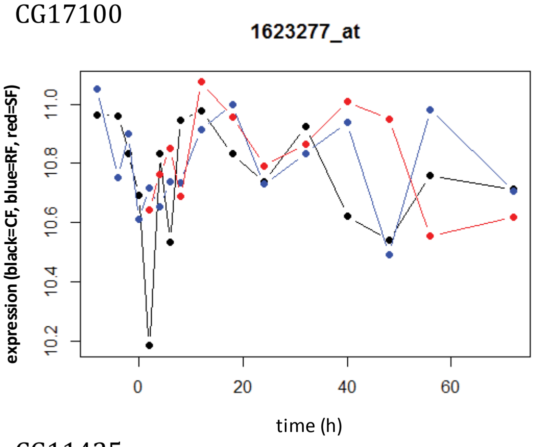

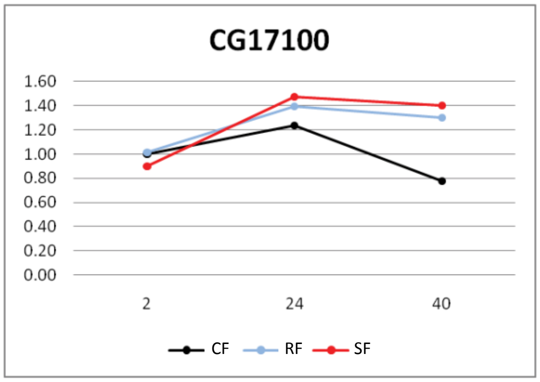

CG11425

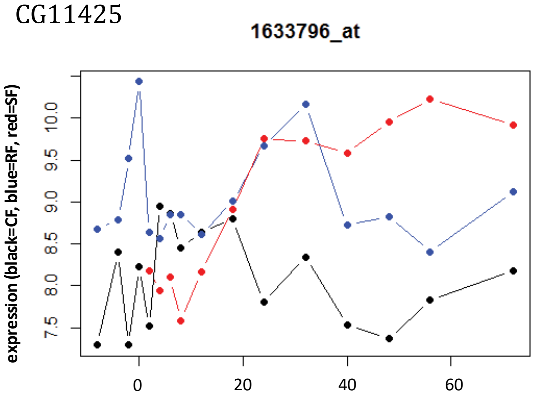

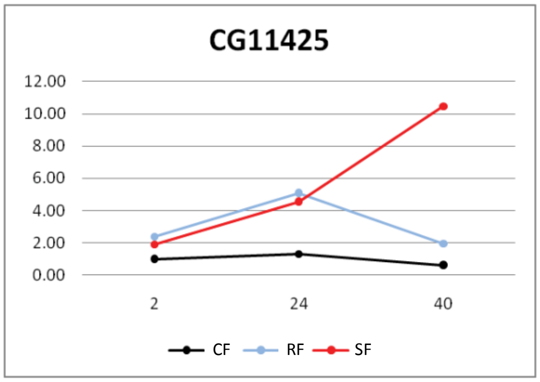

CG3290

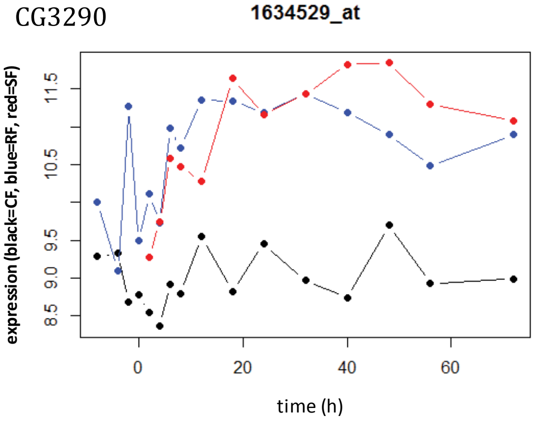

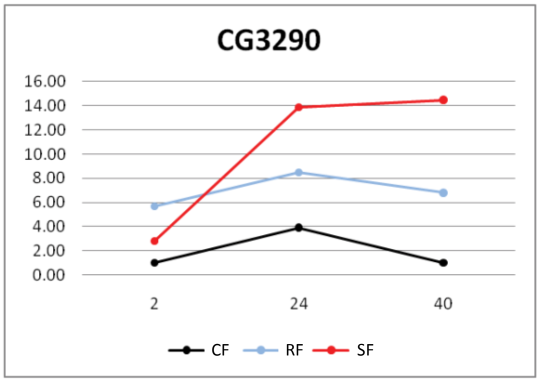

CG10514

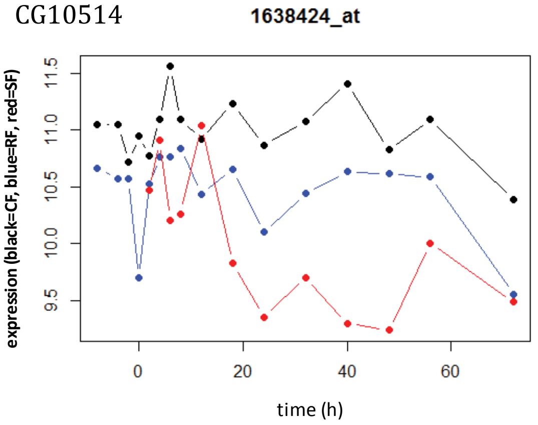

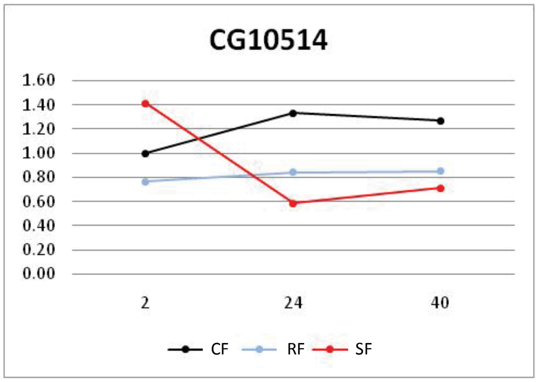

**MICROARRAY**	**qPCR** (normalized to CF 2h)

CG10513

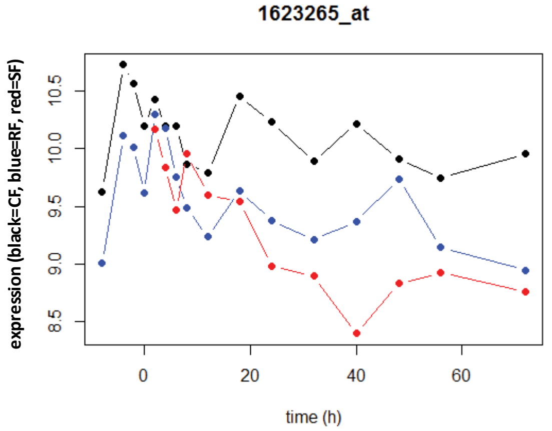

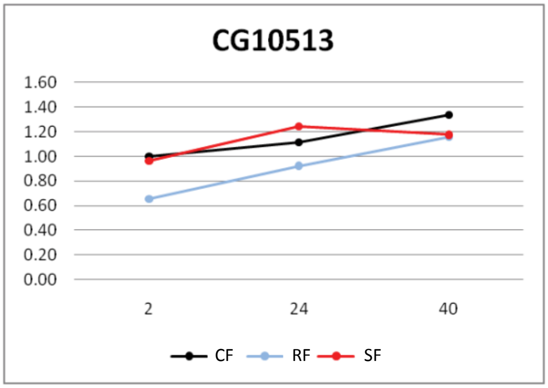

CG8745 1641270_at

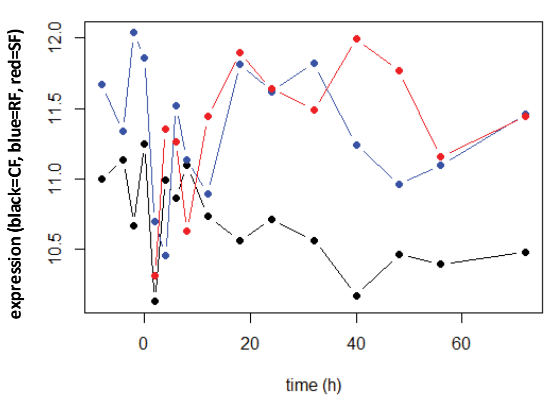

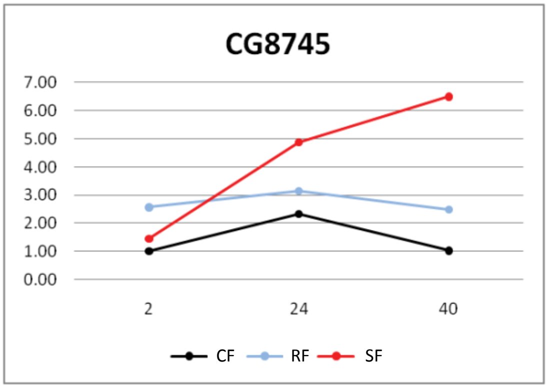

Figure S5
