# Supplementary material for: Dietary switch reveals fast coordinated gene expression changes in Drosophila melanogaster
Source: Aging (Albany NY). 2014 May 14;6(5):355–68. doi: 10.18632/aging.100662 (PMC4069263; doi:10.18632/aging.100662)
Supplement: Supplementary file 2 [file aging-06-355-s002.docx]

**Table S1 – Gene ontologies enriched for down- and up-regulated genes in RF versus CF**

| **GO Term** | | **q value**^¶^ | **# genes** |
| --- | --- | --- | --- |
| **Genes Down-regulated** | **Total genes matched:**240 | | |
| single-organism metabolic process [GO:0044710] | | 3.88E-19 | 95 |
| [oxidation-reduction process [GO:0055114]](http://amigo.geneontology.org/cgi-bin/amigo/term_details?term=GO:0055114) | | 2.78E-14 | 51 |
| [carboxylic acid metabolic process [GO:0019752]](http://amigo.geneontology.org/cgi-bin/amigo/term_details?term=GO:0019752) | | 8.64E-10 | 32 |
| [organic acid metabolic process [GO:0006082]](http://amigo.geneontology.org/cgi-bin/amigo/term_details?term=GO:0006082) | | 9.39E-10 | 33 |
| [oxoacid metabolic process [GO:0043436]](http://amigo.geneontology.org/cgi-bin/amigo/term_details?term=GO:0043436) | | 9.39E-10 | 33 |
| [cellular lipid catabolic process [GO:0044242]](http://amigo.geneontology.org/cgi-bin/amigo/term_details?term=GO:0044242) | | 5.70E-08 | 11 |
| [small molecule metabolic process [GO:0044281]](http://amigo.geneontology.org/cgi-bin/amigo/term_details?term=GO:0044281) | | 6.33E-08 | 47 |
| [fatty acid oxidation [GO:0019395]](http://amigo.geneontology.org/cgi-bin/amigo/term_details?term=GO:0019395) | | 1.69E-07 | 8 |
| [lipid metabolic process [GO:0006629]](http://amigo.geneontology.org/cgi-bin/amigo/term_details?term=GO:0006629) | | 1.85E-07 | 30 |
| [lipid oxidation [GO:0034440]](http://amigo.geneontology.org/cgi-bin/amigo/term_details?term=GO:0034440) | | 3.20E-07 | 8 |
| [carbohydrate metabolic process [GO:0005975]](http://amigo.geneontology.org/cgi-bin/amigo/term_details?term=GO:0005975) | | 6.57E-07 | 27 |
| [fatty acid beta-oxidation [GO:0006635]](http://amigo.geneontology.org/cgi-bin/amigo/term_details?term=GO:0006635) | | 2.86E-06 | 7 |
| [lipid catabolic process [GO:0016042]](http://amigo.geneontology.org/cgi-bin/amigo/term_details?term=GO:0016042) | | 3.07E-06 | 13 |
| [cellular lipid metabolic process [GO:0044255]](http://amigo.geneontology.org/cgi-bin/amigo/term_details?term=GO:0044255) | | 3.87E-06 | 22 |
| [monocarboxylic acid metabolic process [GO:0032787]](http://amigo.geneontology.org/cgi-bin/amigo/term_details?term=GO:0032787) | | 7.64E-06 | 14 |
| [fatty acid catabolic process [GO:0009062]](http://amigo.geneontology.org/cgi-bin/amigo/term_details?term=GO:0009062) | | 8.37E-06 | 7 |
| [fatty acid metabolic process [GO:0006631]](http://amigo.geneontology.org/cgi-bin/amigo/term_details?term=GO:0006631) | | 1.07E-05 | 11 |
| [monocarboxylic acid catabolic process [GO:0072329]](http://amigo.geneontology.org/cgi-bin/amigo/term_details?term=GO:0072329) | | 2.16E-05 | 7 |
| [hexose metabolic process [GO:0019318]](http://amigo.geneontology.org/cgi-bin/amigo/term_details?term=GO:0019318) | | 2.71E-04 | 11 |
| [lipid modification [GO:0030258]](http://amigo.geneontology.org/cgi-bin/amigo/term_details?term=GO:0030258) | | 3.39E-04 | 9 |
| [monosaccharide metabolic process [GO:0005996]](http://amigo.geneontology.org/cgi-bin/amigo/term_details?term=GO:0005996) | | 3.75E-04 | 11 |
| [glucose metabolic process [GO:0006006]](http://amigo.geneontology.org/cgi-bin/amigo/term_details?term=GO:0006006) | | 5.40E-04 | 9 |
| [small molecule catabolic process [GO:0044282]](http://amigo.geneontology.org/cgi-bin/amigo/term_details?term=GO:0044282) | | 0.002948 | 9 |
| [single-organism catabolic process [GO:0044712]](http://amigo.geneontology.org/cgi-bin/amigo/term_details?term=GO:0044712) | | 0.002948 | 9 |
| [pentose-phosphate shunt [GO:0006098]](http://amigo.geneontology.org/cgi-bin/amigo/term_details?term=GO:0006098) | | 0.004829 | 4 |
| [NADPH regeneration [GO:0006740]](http://amigo.geneontology.org/cgi-bin/amigo/term_details?term=GO:0006740) | | 0.004829 | 4 |
| [single-organism carbohydrate metabolic process [GO:0044723]](http://amigo.geneontology.org/cgi-bin/amigo/term_details?term=GO:0044723) | | 0.008316 | 15 |
| [cellular amino acid metabolic process [GO:0006520]](http://amigo.geneontology.org/cgi-bin/amigo/term_details?term=GO:0006520) | | 0.009486 | 16 |
| [determination of adult lifespan [GO:0008340]](http://amigo.geneontology.org/cgi-bin/amigo/term_details?term=GO:0008340) | | 0.01118 | 13 |
| [multicellular organismal aging [GO:0010259]](http://amigo.geneontology.org/cgi-bin/amigo/term_details?term=GO:0010259) | | 0.011591 | 13 |
| [aging [GO:0007568]](http://amigo.geneontology.org/cgi-bin/amigo/term_details?term=GO:0007568) | | 0.014748 | 13 |
| [organic acid catabolic process [GO:0016054]](http://amigo.geneontology.org/cgi-bin/amigo/term_details?term=GO:0016054) | | 0.01591 | 7 |
| [carboxylic acid catabolic process [GO:0046395]](http://amigo.geneontology.org/cgi-bin/amigo/term_details?term=GO:0046395) | | 0.01591 | 7 |
| [metabolic process [GO:0008152]](http://amigo.geneontology.org/cgi-bin/amigo/term_details?term=GO:0008152) | | 0.022329 | 166 |
| [NADP metabolic process [GO:0006739]](http://amigo.geneontology.org/cgi-bin/amigo/term_details?term=GO:0006739) | | 0.022598 | 4 |
| [triglyceride homeostasis [GO:0070328]](http://amigo.geneontology.org/cgi-bin/amigo/term_details?term=GO:0070328) | | 0.022598 | 4 |
| [coenzyme metabolic process [GO:0006732]](http://amigo.geneontology.org/cgi-bin/amigo/term_details?term=GO:0006732) | | 0.023926 | 8 |
| [lipid homeostasis [GO:0055088]](http://amigo.geneontology.org/cgi-bin/amigo/term_details?term=GO:0055088) | | 0.027388 | 5 |
| [acylglycerol homeostasis [GO:0055090]](http://amigo.geneontology.org/cgi-bin/amigo/term_details?term=GO:0055090) | | 0.028022 | 4 |
| [sulfur compound metabolic process [GO:0006790]](http://amigo.geneontology.org/cgi-bin/amigo/term_details?term=GO:0006790) | | 0.028695 | 9 |
| [organonitrogen compound metabolic process [GO:1901564]](http://amigo.geneontology.org/cgi-bin/amigo/term_details?term=GO:1901564) | | 0.036801 | 33 |
| [transmembrane transport [GO:0055085]](http://amigo.geneontology.org/cgi-bin/amigo/term_details?term=GO:0055085) | | 0.041891 | 23 |
|  | |  |  |
| **Genes Up-regulated Total genes matched:**341 | | | |
| electron transport chain [GO:0022900] | | 4.83E-10 | 21 |
| [respiratory electron transport chain [GO:0022904]](http://amigo.geneontology.org/cgi-bin/amigo/term_details?term=GO:0022904) | | 8.83E-10 | 20 |
| [energy derivation by oxidation of organic compounds [GO:0015980]](http://amigo.geneontology.org/cgi-bin/amigo/term_details?term=GO:0015980) | | 3.45E-09 | 24 |
| [cellular respiration [GO:0045333]](http://amigo.geneontology.org/cgi-bin/amigo/term_details?term=GO:0045333) | | 3.49E-09 | 23 |
| [generation of precursor metabolites and energy [GO:0006091]](http://amigo.geneontology.org/cgi-bin/amigo/term_details?term=GO:0006091) | | 1.99E-08 | 26 |
| [mitochondrial ATP synthesis coupled electron transport [GO:0042775]](http://amigo.geneontology.org/cgi-bin/amigo/term_details?term=GO:0042775) | | 2.27E-08 | 17 |
| [ATP synthesis coupled electron transport [GO:0042773]](http://amigo.geneontology.org/cgi-bin/amigo/term_details?term=GO:0042773) | | 4.76E-08 | 17 |
| [oxidative phosphorylation [GO:0006119]](http://amigo.geneontology.org/cgi-bin/amigo/term_details?term=GO:0006119) | | 1.81E-07 | 17 |
| [oxidation-reduction process [GO:0055114]](http://amigo.geneontology.org/cgi-bin/amigo/term_details?term=GO:0055114) | | 1.42E-06 | 47 |
| [proteolysis [GO:0006508]](http://amigo.geneontology.org/cgi-bin/amigo/term_details?term=GO:0006508) | | 2.98E-05 | 54 |
| [single-organism metabolic process [GO:0044710]](http://amigo.geneontology.org/cgi-bin/amigo/term_details?term=GO:0044710) | | 3.11E-05 | 85 |
| [mitochondrial electron transport, NADH to ubiquinone [GO:0006120]](http://amigo.geneontology.org/cgi-bin/amigo/term_details?term=GO:0006120) | | 4.22E-05 | 10 |
| [transmembrane transport [GO:0055085]](http://amigo.geneontology.org/cgi-bin/amigo/term_details?term=GO:0055085) | | 1.03E-04 | 37 |

^¶^ The Benjamini and Hochberg method was used for Multiple Hypothesis Test correction allowing a maximum value cutoff of 0.05

**Table S2 - Additional categories for genes displayed intermediate behaviors**

| **Cat.** | **Behavior** | **# genes** |
| --- | --- | --- |
| **V** | RF = CF  SF = CF  SF = RF | 12092 |
| **VI** | RF ≠ CF  SF = CF  SF = RF | 509 |
| **VII** | RF = CF  SF ≠ CF  SF = RF | 65 |
| **VIII** | RF = CF  SF = CF  SF ≠ RF | 61 |

**Table S3 – Gene ontologies enriched for genes in each of the four main classes of temporal responses to the switch in diet**

| **GO Term** | | **q value**^¶^ | **# genes** |
| --- | --- | --- | --- |
| **Category I: Genes Down-regulated** |  | | |
| single-organism metabolic process [GO:0044710] | | 1.15E-08 | 38 |
| [carboxylic acid metabolic process [GO:0019752]](http://amigo.geneontology.org/cgi-bin/amigo/term_details?term=GO:0019752) | | 3.52E-06 | 16 |
| [oxidation-reduction process [GO:0055114]](http://amigo.geneontology.org/cgi-bin/amigo/term_details?term=GO:0055114) | | 3.91E-06 | 21 |
| [organic acid metabolic process [GO:0006082]](http://amigo.geneontology.org/cgi-bin/amigo/term_details?term=GO:0006082) | | 5.14E-06 | 16 |
| [oxoacid metabolic process [GO:0043436]](http://amigo.geneontology.org/cgi-bin/amigo/term_details?term=GO:0043436) | | 5.14E-06 | 16 |
| [small molecule metabolic process [GO:0044281]](http://amigo.geneontology.org/cgi-bin/amigo/term_details?term=GO:0044281) | | 1.20E-04 | 21 |
| [monosaccharide metabolic process [GO:0005996]](http://amigo.geneontology.org/cgi-bin/amigo/term_details?term=GO:0005996) | | 0.015809 | 6 |
| [hexose metabolic process [GO:0019318]](http://amigo.geneontology.org/cgi-bin/amigo/term_details?term=GO:0019318) | | 0.017139 | 6 |
| [carbohydrate metabolic process [GO:0005975]](http://amigo.geneontology.org/cgi-bin/amigo/term_details?term=GO:0005975) | | 0.017893 | 11 |
| [pentose-phosphate shunt [GO:0006098]](http://amigo.geneontology.org/cgi-bin/amigo/term_details?term=GO:0006098) | | 0.01837 | 3 |
| [NADPH regeneration [GO:0006740]](http://amigo.geneontology.org/cgi-bin/amigo/term_details?term=GO:0006740) | | 0.01837 | 3 |
| [glucose metabolic process [GO:0006006]](http://amigo.geneontology.org/cgi-bin/amigo/term_details?term=GO:0006006) | | 0.022655 | 5 |
| [monocarboxylic acid metabolic process [GO:0032787]](http://amigo.geneontology.org/cgi-bin/amigo/term_details?term=GO:0032787) | | 0.035707 | 6 |
| [NADP metabolic process [GO:0006739]](http://amigo.geneontology.org/cgi-bin/amigo/term_details?term=GO:0006739) | | 0.0453 | 3 |
| **Category I: Genes Up-regulated** | |  | |

| Number of GO-enriched genes: 0 |  |  |
| --- | --- | --- |

| **Category II: Genes Down-regulated** | |  | | |
| --- | --- | --- | --- | --- |
| cellular lipid catabolic process [GO:0044242] | | | 0.00944 | 4 |
| [lipid catabolic process [GO:0016042]](http://amigo.geneontology.org/cgi-bin/amigo/term_details?term=GO:0016042) | | | 0.017273 | 5 |
| [lipid metabolic process [GO:0006629]](http://amigo.geneontology.org/cgi-bin/amigo/term_details?term=GO:0006629) | | | 0.02063 | 8 |
| **Category II: Genes Up-regulated** |  | | | |
| electron transport chain [GO:0022900] | | | 2.09E-07 | 11 |
| [respiratory electron transport chain [GO:0022904]](http://amigo.geneontology.org/cgi-bin/amigo/term_details?term=GO:0022904) | | | 2.31E-07 | 11 |
| [mitochondrial ATP synthesis coupled electron transport [GO:0042775]](http://amigo.geneontology.org/cgi-bin/amigo/term_details?term=GO:0042775) | | | 3.18E-07 | 10 |
| [ATP synthesis coupled electron transport [GO:0042773]](http://amigo.geneontology.org/cgi-bin/amigo/term_details?term=GO:0042773) | | | 4.50E-07 | 10 |
| [oxidative phosphorylation [GO:0006119]](http://amigo.geneontology.org/cgi-bin/amigo/term_details?term=GO:0006119) | | | 7.20E-07 | 10 |
| [energy derivation by oxidation of organic compounds [GO:0015980]](http://amigo.geneontology.org/cgi-bin/amigo/term_details?term=GO:0015980) | | | 8.05E-07 | 12 |
| [cellular respiration [GO:0045333]](http://amigo.geneontology.org/cgi-bin/amigo/term_details?term=GO:0045333) | | | 2.60E-06 | 11 |
| [proteolysis [GO:0006508]](http://amigo.geneontology.org/cgi-bin/amigo/term_details?term=GO:0006508) | | | 8.72E-06 | 24 |
| [generation of precursor metabolites and energy [GO:0006091]](http://amigo.geneontology.org/cgi-bin/amigo/term_details?term=GO:0006091) | | | 8.75E-06 | 12 |
| [mitochondrial electron transport, NADH to ubiquinone [GO:0006120]](http://amigo.geneontology.org/cgi-bin/amigo/term_details?term=GO:0006120) | | | 8.99E-06 | 7 |
| [oxidation-reduction process [GO:0055114]](http://amigo.geneontology.org/cgi-bin/amigo/term_details?term=GO:0055114) | | | 0.009761 | 16 |
| **Category III: Genes Down-regulated** |  | | | |

| Number of GO-enriched genes: 0 |  |  |
| --- | --- | --- |

| **Category III: Genes Up-regulated** |  | | | |
| --- | --- | --- | --- | --- |
| Number of GO-enriched genes: 0 | |  | | |
| **Category IV: Genes Down-regulated** |  | | | |
| transmembrane transport [GO:0055085] | | | 0.016796 | 5 |
| **Category IV: Genes Up-regulated** |  | | | |
| Number of GO-enriched genes: 0 |  | | | |

^¶^ The Benjamini and Hochberg method was used for Multiple Hypothesis Test correction allowing a maximum value cutoff of 0.05
